# Global profiling of the proteome, phosphoproteome, and N-glycoproteome of protoscoleces and adult worms of *Echinococcus granulosus*

**DOI:** 10.3389/fvets.2023.1275486

**Published:** 2023-11-02

**Authors:** Zhengrong Wang, Xinyue Jia, Jing Ma, Yanyan Zhang, Yan Sun, Xinwen Bo

**Affiliations:** ^1^State Key Laboratory of Sheep Genetic Improvement and Healthy Production, Xinjiang Academy of Agricultural and Reclamation Science, Shihezi, China; ^2^Institute of Animal Husbandry and Veterinary Medicine, Xinjiang Academy of Agricultural and Reclamation Science, Shihezi, China; ^3^College of Animal Science and Technology, Shihezi University, Shihezi, China

**Keywords:** *Echinococcus granulosus*, protoscoleces, adult worm, proteome, phosphoproteome, N-glycoproteome

## Abstract

**Introduction:**

Cystic echinococcosis (CE) is a chronic zoonosis caused by infection with the metacestode of the *Echinococcus granulosus.* A unique characteristic of *E. granulosus* protoscolex (PSC) is their ability to develop bidirectionally into an adult worm in the definitive host or a secondary hydatid cyst in the intermediate host. Furthermore, cestodes have a complex life cycle involving different developmental stages; however, the mechanisms underlying this development remain unknown. Several studies have demonstrated that certain matrix proteins undergo posttranslational modifications (PTMs), including phosphorylation and glycosylation, which have important regulatory effects on their functional properties.

**Methods:**

Systematic analyses of the proteome, phosphorylated modified proteome, and glycosylated modified proteome of protoscoleces (PSCs) and adult worms were performed using a proteomic strategy. Data are available via ProteomeXchange with identifier PXD043166.

**Results:**

In total, 6,407 phosphorylation sites and 1757 proteins were quantified. Of these, 2032 phosphorylation sites and 770 proteins were upregulated, and 2,993 phosphorylation sites and 1,217 proteins were downregulated in adult worms compared to PSCs. A total of 612 N-glycosylation sites were identified in the 392 N-glycoproteins. Of these, 355 N-glycosylation sites and 212 N-glycoproteins were quantified. Of these, 90 N-glycosylation sites and 64 N-glycoproteins were upregulated, and 171 N-glycosylation sites and 126 N-glycoproteins were downregulated in adult worms compared to PSCs. GO enrichment analysis indicated that the differentially expressed phosphoproteins were mainly enriched in the regulation of oxidoreduction coenzyme metabolic processes, myelin sheath, and RNA helicase activity, whereas the differentially expressed N-glycoproteins were enriched in the cellular response to unfolded proteins, endoplasmic reticulum lumen, and nucleic acid binding. KEGG enrichment analysis indicated that the differently expressed phosphoproteins were mainly enriched in RNA transport, hypertrophic cardiomyopathy (HCM), glycolysis/gluconeogenesis, HIF-1 signaling pathway and pyruvate metabolism. Differentially expressed N-glycoproteins were enriched in the PI3K-Akt signaling pathway, ECM-receptor interactions, and protein processing in the endoplasmic reticulum.

**Discussion:**

To our knowledge, this study is the first global phosphoproteomic and N-glycoproteomic analysis of *E. granulosus*, which provides valuable information on the expression characteristics of *E. granulosus* and provides a new perspective to elucidate the role of protein phosphorylation and N-glycosylation in the development of *E. granulosus*.

## 1. Introduction

*E. granulosus* is one of the main causes of CE, a cosmopolitan zoonotic parasitic disease that considerably impacts public sanitary systems and regional economies (1). Globally, CE occurs mainly in South America, Asia, Western and Central Europe, Australia, and North Africa (2). In China, CE is primarily distributed in Xinjiang, Qinghai, Xizang and Ningxia (3, 4). Carnivorous animals (usually dogs) are definitive hosts of *E. granulosus.* Adult cestodes of *E. granulosus* live within the small intestine of their definitive host and produce and release eggs into the environment via feces. Humans can accidentally acquire the infection by ingesting eggs shed by the definitive hosts. Upon infection, metacestodes proliferate asexually in organs (primarily the liver), causing space-occupying lesions, organ malfunction, and even death (5). To date, the regulatory mechanisms of *E. granulosus* growth and development are poorly understood.

The significance of PTMs in proteins derived from eukaryotic organisms extends beyond the exploration of fundamental biological principles and encompasses the potential for the advancement of biotechnological applications. Moreover, phosphorylation and N-glycosylation are the two most important PTMs in eukaryotic organisms (6). Protein phosphorylation is typically the initial cascade of protein alterations in response to intracellular and extracellular signaling and plays a crucial role in regulating essential physiological processes, including growth signal response, cell cycle progression, and cell stress response (7). The reversible process of protein phosphorylation significantly influences a multitude of biological processes. Certain apicomplexan parasites utilize this mechanism to manipulate host cells effectively (8, 9). N-glycosylation of proteins occurs in the endoplasmic reticulum (ER) and undergoes further modifications in the Golgi apparatus (10). Most proteins that participate in various physiological functions undergo N-glycosylation. These functions include lymphocyte activation, apoptosis, antigen recognition and clearance, cell adhesion, signal transduction, and endocytosis (11). The investigation of PTMs in helminth parasites, including *E. granulosus*, remains a relatively unexplored area of research. Although some studies have been published, the number of PTMs identified in *E. granulosus* proteins is limited, with amidation (12), N-terminal acetylation (13), and N- and O-glycosylation (14) being the most commonly observed modifications, primarily within the PSC stage. Furthermore, geranyl-geranylation (15) and S-farnesylation (16) have been detected in the closely related cestode parasite *E. multilocularis*, whereas mannosylation has been reported in *Taenia solium* proteins (17). Recent studies have determined that 22 potential PTMs are predominantly present in the tegumental proteins of *E. granulosus* PSCs. Of these, nine were identified as having a high degree of certainty (18). However, this type of PTM remains poorly understood in the different developmental stages of *E. granulosus*, including the PSC, adult worms, eggs, and oncosphere. In this study, we employed high-throughput liquid chromatography–tandem mass spectrometry (LC-MS/MS) to investigate the phosphorylation and N-glycosylation of *E. granulosus* as well as their expression patterns in PSCs and adult worms. Our findings substantially augment the existing understanding of phosphorylation and N-glycosylation in *E. granulosus* and offer a comprehensive overview and discussion of the current knowledge of these processes. Furthermore, our results can potentially inform the development of novel intervention strategies for controlling CE.

## 2. Materials and methods

### 2.1. Parasite preparation

Hydatid cysts were collected from the livers of naturally infected sheep from an abattoir in Urumqi, Xinjiang Province, China. The PSCs were aspirated aseptically from hydatid cysts and sedimented at 1,000 × *g* for 15 min and digested by pepsin (19). Then the G1 genotype of the *E. granulosus* was identified as previously described (20). Once the genotype was identified, the PSCs were stored at −80°C prior to use. *E. granulosus* adult worms with four segments were acquired from an 8-month-old canine after a 28-day period of artificial infection through oral gavage with 200,000 PSCs, as previously described (21).

### 2.2. Methods

#### 2.2.1. Protein extraction trypsin digestion

The sample was ground with liquid nitrogen into cell powder and then transferred to a 5-ml centrifuge tube. After that, four volumes of lysis buffer (8 M urea, 1% protease inhibitor cocktail) were added to the cell powder, followed by sonication three times on ice using a high-intensity ultrasonic processor (Scientz). The remaining debris was removed by centrifugation at 12,000 × *g* at 4°C for 10 min. Finally, the supernatant was collected, and the protein concentration was determined using a BCA kit according to the manufacturer’s instructions.

For digestion, the protein solution was reduced with 5 mM dithiothreitol for 30 min at 56°C and alkylated with 11 mM iodoacetamide for 15 min at room temperature in the darkness. The protein sample was then diluted by adding 100 mM TEAB to a urea concentration of less than 2 M. Finally, trypsin was added at a 1:50 trypsin-to-protein mass ratio for the first digestion overnight and a 1:100 trypsin-to-protein mass ratio for the second 4 h digestion. Finally, the peptides were desalted using a C18 SPE column.

#### 2.2.2. Affinity enrichment

##### 2.2.2.1. Bio-material-based PTM enrichment (for phosphorylation)

Peptide mixtures were first incubated with IMAC microsphere suspension with vibration in loading buffer (50% acetonitrile/0.5% acetic acid). To remove the non-specifically adsorbed peptides, the IMAC microspheres were washed sequentially with 50% acetonitrile/0.5% acetic acid and 30% acetonitrile/0.1% trifluoroacetic acid. An elution buffer containing 10% NH_4_OH was added to elute the enriched phosphopeptides by vibration. The supernatant containing the phosphopeptides was collected and lyophilized for LC-MS/MS analysis.

##### 2.2.2.2. Bio-material-based PTM enrichment (for N-glycosylation)

Tryptic peptides were re-dissolved in 200 μl washing buffer (80% ACN, 5% TFA), loaded onto the column, and washed with washing buffer thrice. Glycopeptides were eluted with 0.1% TFA, 50 mM ammonium bicarbonate, and 50% ACN twice. The eluted glycopeptides were dried in the Speedvac and re-dissolved with 50 μl of 50 mM ammonium bicarbonate solution dissolved in H_2_O^18^. After adding 2 μl of PNGase F glycosidase, the digestion was performed at 37°C overnight. Finally, the deglycopeptides were desalted using C18 Zip Tips, according to the manufacturer’s instructions, and dried for further MS analysis.

### 2.3. LC–MS/MS analysis

The tryptic peptides were dissolved in solvent A (0.1% formic acid, 2% acetonitrile/in water) and directly loaded onto a homemade reverse-phase analytical column (25 cm length, 75/100 μm i.d.). Peptides were separated with a gradient from 6 to 24% solvent B (0.1% formic acid in acetonitrile) over 70 min, 24 to 35% in 14 min, increasing to 80% in 3 min, then maintained at 80% for the last 3 min, all at a constant flow rate of 450 nL/min on a nanoElute UHPLC system (Bruker Daltonics).

The peptides were subjected to capillary source analysis and timsTOF Pro (Bruker Daltonics) mass spectrometry. The applied electrospray voltage was 1.60 kV. Precursors and fragments were analyzed using a TOF detector with an MS/MS scan range of 100–1700 m/z. The timsTOF Pro was operated in the parallel accumulation serial fragmentation (PASEF) mode. Precursors with charge states of 0–5 were selected for fragmentation, and 10 PASEF-MS/MS scans were acquired per cycle. The dynamic exclusion was set at 30 s.

### 2.4. Database search

The resulting MS/MS data were processed using MaxQuant search engine (v.1.6.15.0). Tandem mass spectra were searched against the NCBI database (*E. granulosus*, 11,279 entries) concatenated with reverse decoy database. Trypsin/P was specified as cleavage enzyme allowing up to 2 missing cleavages. The mass tolerance for precursor ions was set as 20 ppm in First search and 20 ppm in Main search, and the mass tolerance for fragment ions was set as 20 ppm. Carbamidomethyl on Cys was specified as fixed modification. Acetylation on protein N-terminal and oxidation on Met were specified as variable modifications. Additionally, phosphorylation on Ser, Thr, Tyr were specified as variable modifications for the searching of phosphopeptides and deamidation of aspartyl amine (^18^O) were specified as variable modifications for the searching of N-glycopeptides. FDR was adjusted to <1%.

### 2.5. Go and KEGG pathway annotation

Proteins were classified by GO annotation into three categories: biological processes, cellular compartments, and molecular functions. For each category, a two-tailed Fisher’s exact test was used to test the enrichment of differentially expressed proteins against all identified proteins. Statistical GO with a corrected *p*-value < 0.05 was considered significant. Enrichment of pathway analysis: The Encyclopedia of Genes and Genomes (KEGG) database was used to identify enriched pathways using a two-tailed Fisher’s exact test to test the enrichment of the differentially expressed proteins against all identified proteins. The pathway with a corrected *p*-value < 0.05 was considered significant. These pathways were classified into hierarchical categories according to the KEGG website.

Enrichment of protein domain analysis: For each category of protein, the InterPro (a resource that provides functional analysis of protein sequences by classifying them into families and predicting the presence of domains and important sites) database was researched, and a two-tailed Fisher’s exact test was employed to test the enrichment of the differentially expressed proteins against all identified proteins. Protein domains with a corrected *p*-value < 0.05 were considered significant.

### 2.6. Domain annotation

The identified protein domain functional descriptions were annotated using InterProScan (a sequence analysis application) based on the protein sequence alignment method and the InterPro domain database. InterPro^1^ is a database that integrates diverse information about protein families, domains, and functional sites and is freely available to the public via Web-based interfaces and services. Central to the database are diagnostic models, known as signatures, against which protein sequences can be searched to determine their potential functions. InterPro is useful in the large-scale analysis of whole genomes and meta-genomes, as well as in characterizing individual protein sequences.

### 2.7. Protein–protein interaction network

All differentially expressed protein database accessions or sequences were searched against the STRING database version 11.0 for protein–protein interactions. Only interactions between proteins belonging to the searched dataset were selected, excluding external candidates. STRING defines a metric called “confidence score” to define interaction confidence; we fetched all interactions that had a confidence score ≥ 0.7 (high confidence). Interaction network form STRING was visualized in the R package “networkD3.”

## 3. Results

### 3.1. Identification of the differentially expressed proteins in the protoscolex and adult worms of *Echinococcus granulosus*

**Figure 1:**
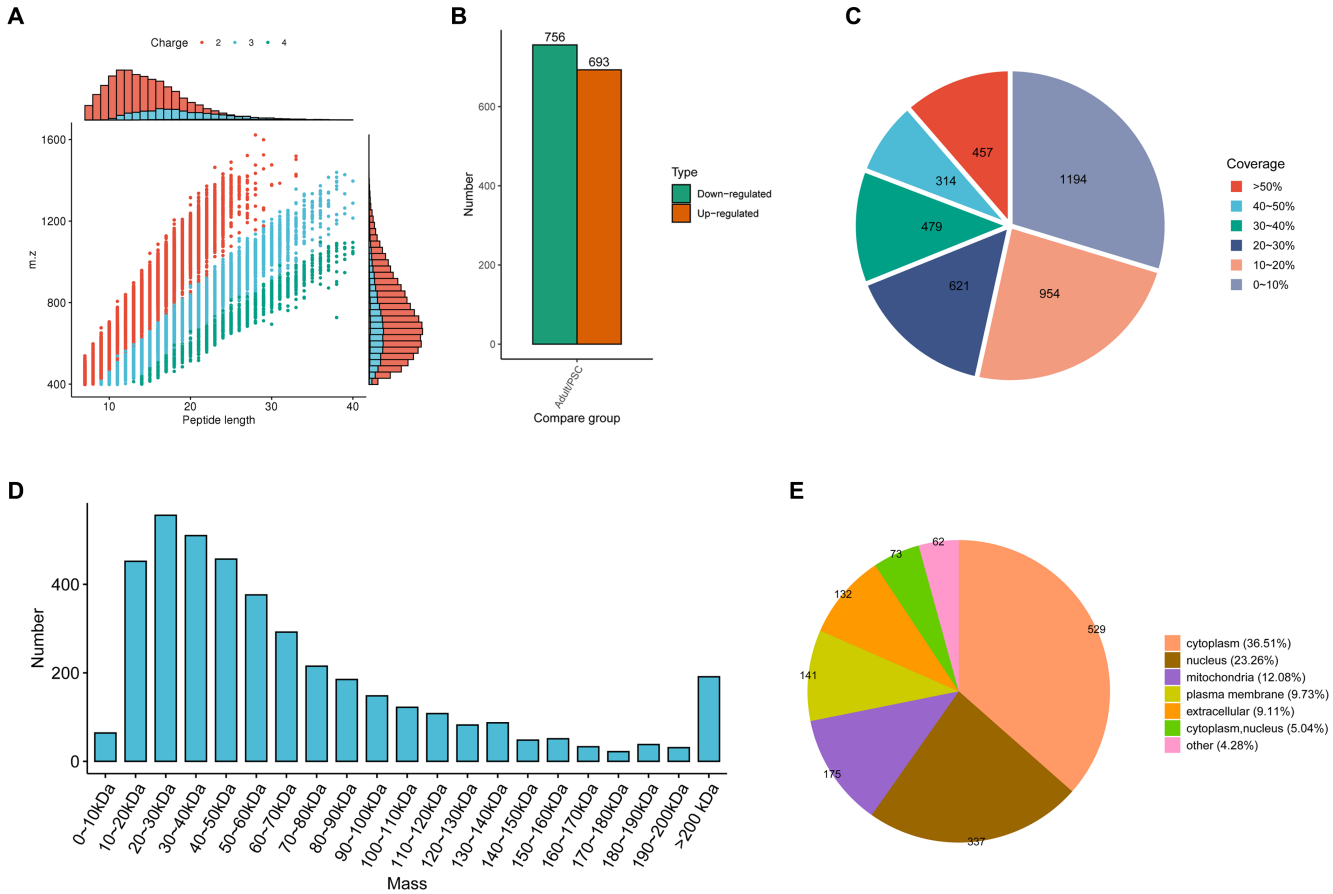
Characteristics of the identified differentially expressed proteins in the protoscolex and adult worms of *E. granulosus*. **(A)** The lengths of most of the identified peptides. **(B)** The differentially expressed pattern of the identified proteins in the protoscolex and adult worms of *E. granulosus*. **(C)** The sequence coverage of identified proteins. **(D)** The theoretical molecular weight of the identified proteins. **(E)** The subcellular localization of the differentially expressed proteins.

In the present study, 34,659 peptides were identified with high precision in the protoscolex and adult worms of *E. granulosus*. Most of the identified peptides were 7–20 amino acid residues long (Figure 1A). After clearing the repeat sequences, 33,866 unique peptides were identified. Simultaneously, we identified 3,949 proteins in the protoscolex and adult worms of *E. granulosus,* of which 2,272 were quantifiable (Supplementary Table 1). By comparing adult worms to the protoscolex, 693 proteins were upregulated, and 756 were downregulated (Figure 1B). The sequence coverage of identified proteins is shown in Figure 1C; 70.29% of the sequence coverage was higher than 10%. The theoretical molecular weight of the identified proteins was calculated, and most of the proteins (58.07%) were distributed between 10 and 60 kDa (Figure 1D). The differentially expressed proteins in protoscolex, and adult worms were detected in the cytoplasm (36.51%), nucleus (23.26%), mitochondria (12.08%), plasma membrane (9.73%), extracellular (9.11%), cytoplasm and nucleus (5.04%), and other (4.28%; Figure 1E).

### 3.2. Differentially phosphorylated proteins in the protoscolex and adult worms of *Echinococcus granulosus*

**Figure 2:**
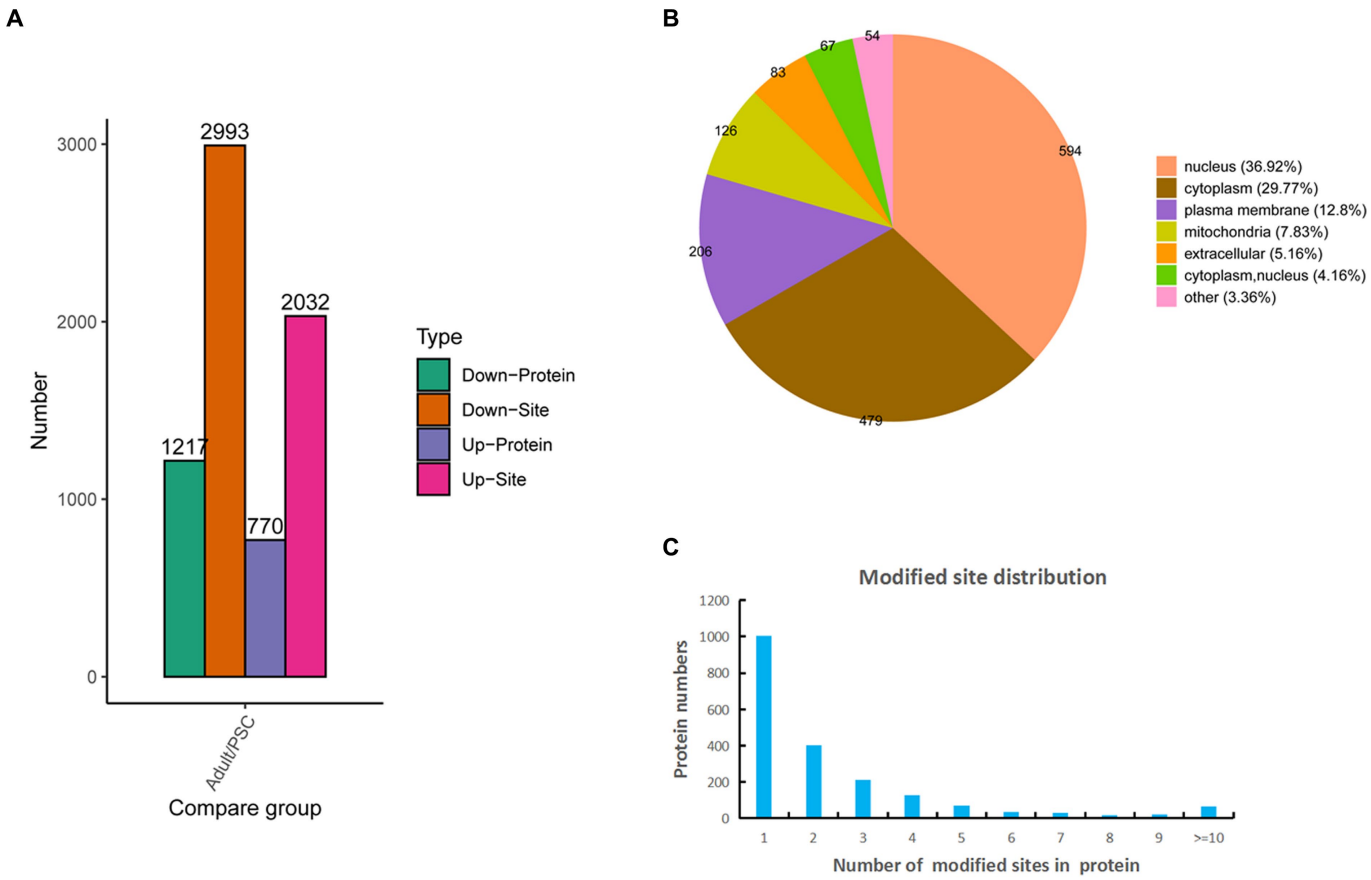
Characteristics of the identified differentially expressed phosphorylated proteins in the protoscolex and adult worms of *E. granulosus*. **(A)** The differentially expressed pattern of the identified phosphorylated proteins in the protoscolex and adult worms of *E. granulosus*. **(B)** The subcellular localization of the differentially expressed phosphorylated proteins. **(C)** Distribution of the phosphorylation site counts of identified differentially expressed phosphorylated proteins.

Based on the mass spectrometry data, 10,327 phosphopeptides were identified from a database search of 16,313 peptides and 3,264 proteins, of which 6,407 sites and 1,757 proteins were quantifiable (Supplementary Table 2). Of these, 2032 sites and 770 proteins were upregulated, and 2,993 sites and 1,217 proteins were downregulated (Figure 2A). Identification of subcellular locations indicated that 36.92% of differentially phosphorylated proteins in protoscolex and adult worms were located in the nucleus, 29.77% in the cytoplasm, 12.8% in the plasma membrane, 7.83% in the mitochondria, and 5.16% in the extracellular (Figure 2B). Statistical analysis of the identified *E. granulosus* phosphorylation sites revealed that phosphorylated modification of *E. granulosus* proteins mainly occurred on serine (S), threonine (T), and tyrosine (Y) residues at ratios of 4,394:544:87 (or 87%:11%:2%), respectively. Most of the identified phosphoproteins (1,002, accounting for 50.25%) in *E. granulosus* only carried a single phosphorylation site, 402 (20.35%) identified phosphoproteins carried two phosphorylation sites, 214 (10.26%) identified phosphoproteins carried three phosphorylation sites, 128 (6.52%) identified phosphoproteins carried four phosphorylation sites, and 241 (12.22%) identified phosphoproteins have multiple phosphorylation sites (>4; Figure 2C). Among these, the galectin domain-containing protein contained the highest number of phosphorylation sites (22).

### 3.3. Differentially N-glycosylated proteins in the protoscolex and adult worms of *Echinococcus granulosus*

**Figure 3:**
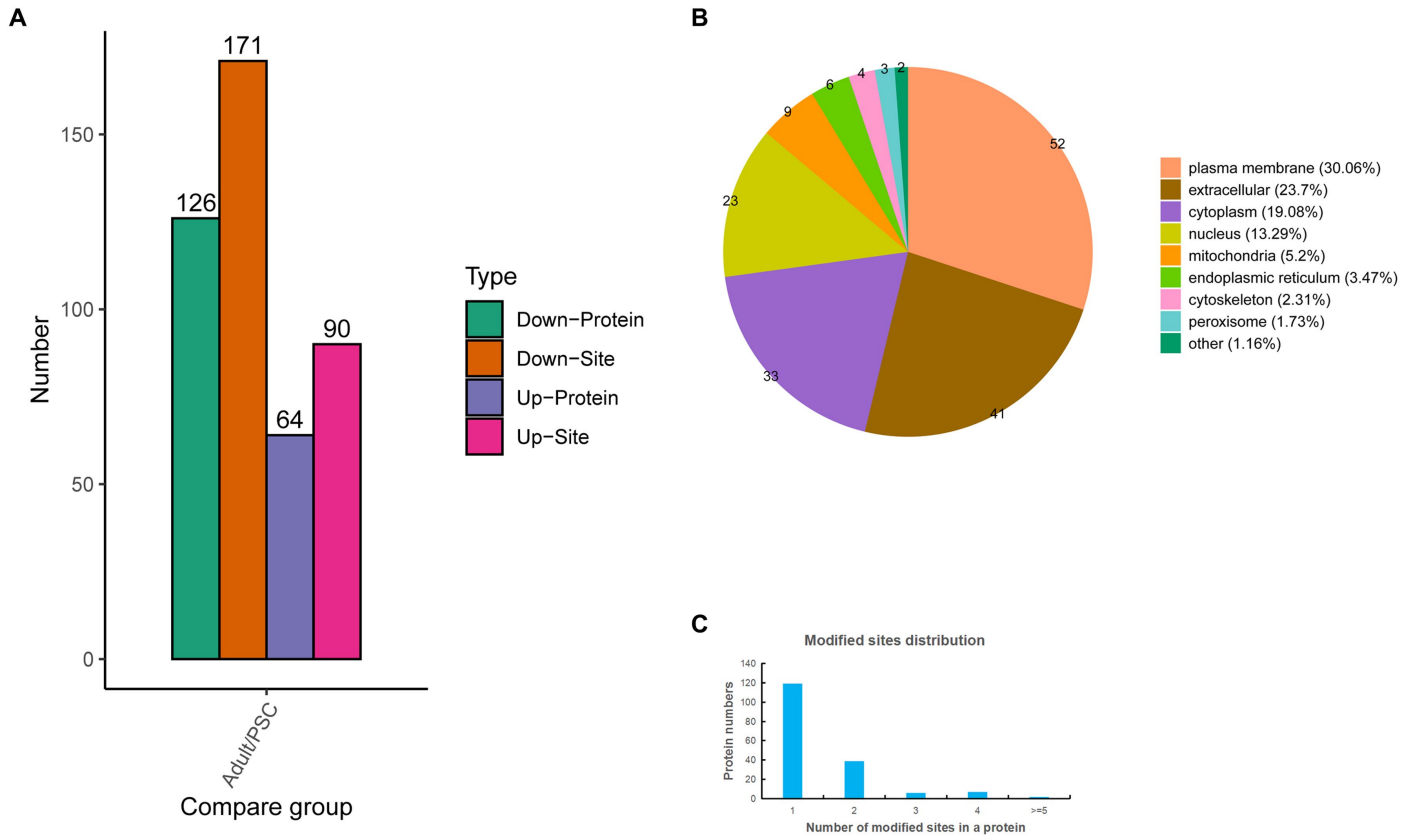
Characteristics of the identified differentially expressed N-glycosylated proteins in the protoscolex and adult worms of *E. granulosus*. **(A)** The differentially expressed pattern of the identified N-glycosylated proteins in the protoscolex and adult worms of *E. granulosus*. **(B)** The subcellular localization of the differentially expressed N-glycosylated proteins. **(C)** Distribution of the N-glycosylation site counts of identified differentially expressed N-glycosylated proteins.

To gain further insight into the differences in N-glycosylation between the protoscolex and adult worms of *E. granulosus.* The glycopeptides were enriched using a HILIC microcolumn and analyzed by LC–MS/MS. A total of 576 unique glycopeptides were obtained, and 612 N-glycosylation sites within 392 N-glycoproteins were identified. Of these, 355 N-glycosylation sites and 212 N-glycoproteins were quantified (Supplementary Table 3). Of these, 90 N-glycosylation sites and 64 N-glycoproteins were upregulated, and 171 N-glycosylation sites and 126 N-glycoproteins were downregulated (Figure 3A). Identification of subcellular locations indicated that 30.06% of differentially N-glycoproteins in protoscolex and adult worms were located in the plasma membrane, 23.7% in the extracellular, 19.08% in the cytoplasm, 13.29% in the nucleus, 5.2% in the mitochondria, and 8.67% in others (Figure 3B). Most of the identified N-glycoproteins (119, accounting for 68.79%) in *E. granulosus* only carried a single N-glycosylation site, 39 (22.54%) identified N-glycoproteins carried two N-glycosylation sites, six (3.47%) identified N-glycoproteins carried three N-glycosylation sites, and nine (5.2%) identified N-glycoproteins had multiple N-glycosylation sites (>4; Figure 3C). The basement membrane-specific heparan sulfate proteoglycan core protein contained the most N-glycosylation sites (13).

### 3.4. Panorama proteome of the protoscolex and adult worms of *Echinococcus granulosus*

**Figure 4:**
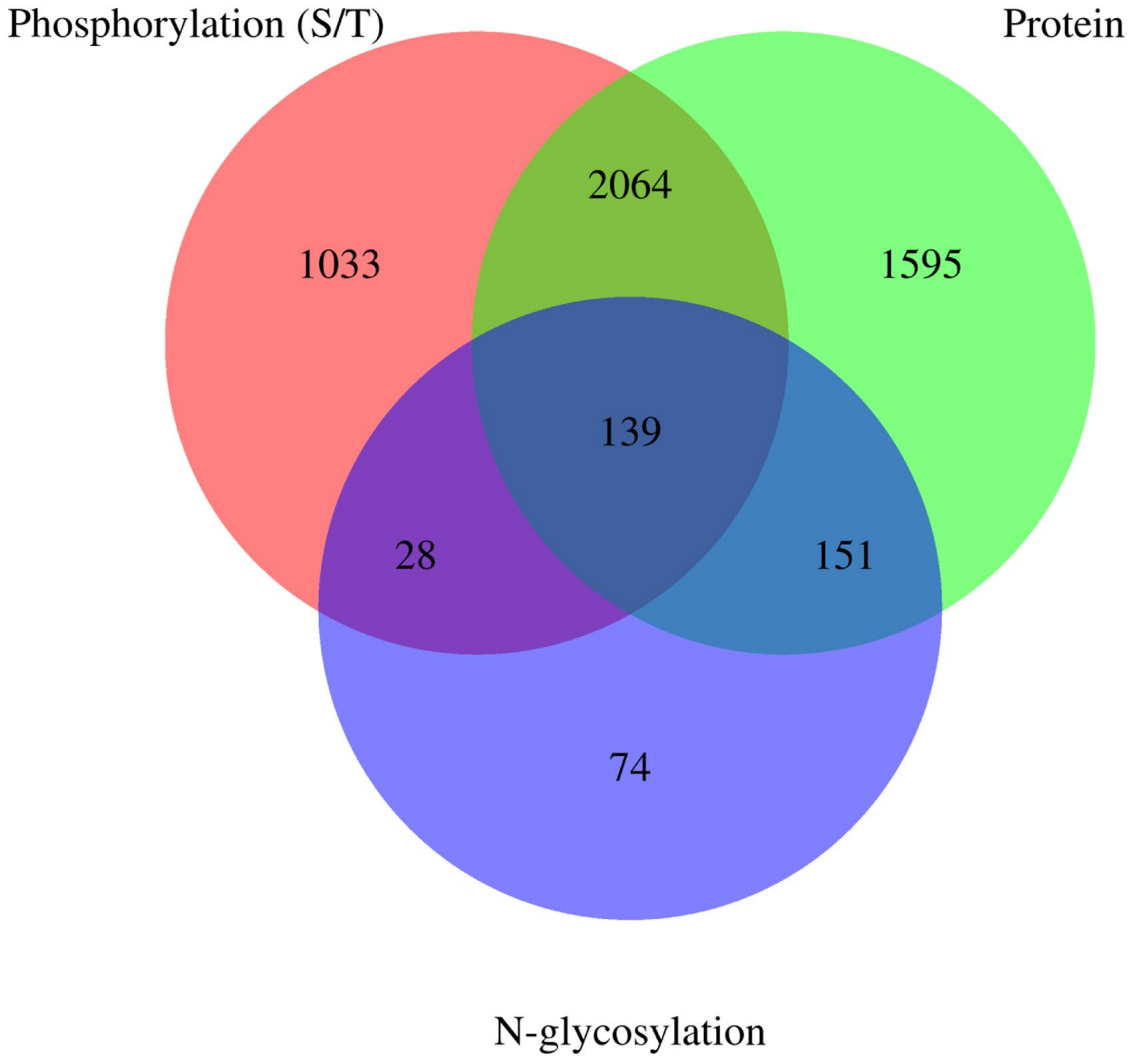
Venn diagrams of *E. granulosus* proteome, phosphoproteome, and N-glycoproteome. Protein: The numuber of identified proteins from *E. granulosus*. Phosphorylation (S/T): The numuber of identified phosphorylation (S/T) proteins from *E. granulosus*. N-glycosylation: The numuber of identified N-glycosylation proteins from *E. granulosus*.

Venn diagrams of *E. granulosus* proteome, phosphoproteome, and N-glycoproteome are shown in Figure 4. A total of 3,949 proteins were identified in the holoproteome, whereas 3,264 and 392 proteins were identified with the addition of phosphoproteins and N-glycoproteins, respectively. This study identified 67.49% of phosphoproteins and 57.40% of N-glycoproteins in the holoproteome. These results indicated that the abundance of phosphoproteins is inferior to that of N-glycoproteins, which require a large amount of affinity enrichment before the determination of phosphoproteins by MS. In addition, 167 proteins, including calreticulin, TPx, tetraspanin, and malate dehydrogenase, were modified by both phosphorylation and N-glycosylation. Therefore, these proteins are more worthy of investigation than single-type modifications.

### 3.5. Motif analysis of phosphorylated proteins in the protoscolex and adult worms of *Echinococcus granulosus*

**Figure 5:**
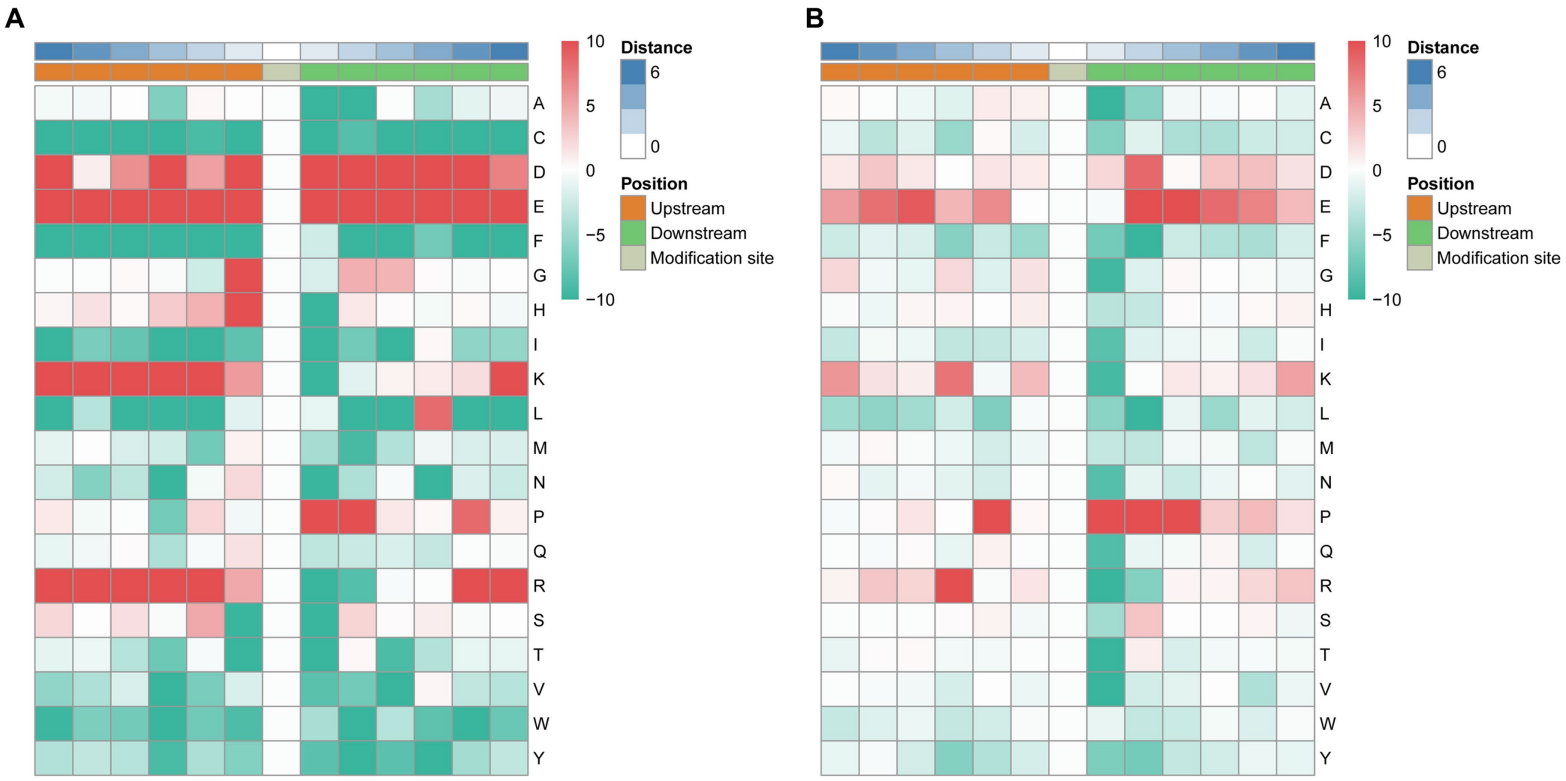
Motif enrichment of phosphorylation sites of the identified phosphopeptides. The MoMo software and hierarchical cluster analysis were used to study the phosphorylation sites from six amino acids upstream to six amino acids downstream of the flanking sequences. **(A)** Motif enrichment of phosphorylation sites in the S motif model. **(B)** Motif enrichment of phosphorylation sites in the T motif model.

To analyze the characteristic sequences of the phosphorylation sites and their enrichment, MoMo software and hierarchical cluster analysis were used to study the phosphorylation sites from six amino acids upstream to six amino acids downstream of the flanking sequences. In the S motif model, the frequency of aspartic acid (D) residue at positions −6, −3, −1, +1 to +5 was highest, glutamic acid (E) residue was enriched at −6 to +6 positions, glycine (G) and histidine (H) residue was mainly enriched at the −1 position, lysine (K) residue was enriched at −6 to −2, +6 positions, leucine (L) was mainly enriched at the +4 position, proline (P) was enriched at +1, +2, +5 positions, arginine (R) was mainly enriched at −6 to −2, +5, +6 positions (Figure 5A). In the T. motif model, aspartic acid (D) was enriched at position +2, glutamic acid (E) was enriched at −5, −4, +2 to +5 positions, proline (P) was enriched at −2, +1 to +3 positions, and arginine (R) was mainly enriched at the −3 position (Figure 5B). In total, 73 motifs were identified, and the enrichment statistical results showed that the sequence “X-S-X” occurred at the highest frequency (Supplementary Table 4).

### 3.6. Motif analysis of N-glycosylated proteins in the protoscolex and adult worms of *Echinococcus granulosus*

**Figure 6:**
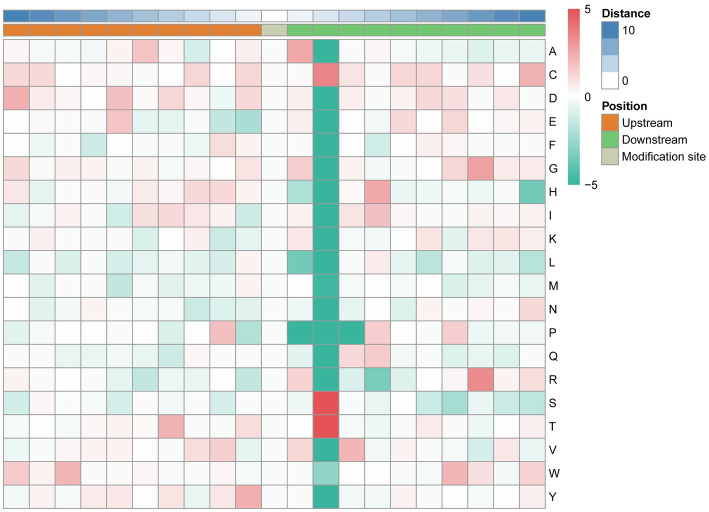
Motif enrichment of N-glycosylation sites of the identified N-glycopeptides. The MoMo software and hierarchical cluster analysis were used to study the N-glycosylation sites from 10 amino acids upstream to 10 amino acids downstream of the flanking sequences.

The MoMo software and hierarchical cluster analysis were used to study the N-glycosylation sites from 10 amino acids upstream to 10 amino acids downstream of the flanking sequences. The frequency of cysteine (C) residue at position +2, arginine (R) residue was mainly enriched at the +8 position, and serine and threonine residues were mainly enriched at the +2 position. Aspartic acid (D), glutamic acid (E), phenylalanine (F), lysine (K), leucine (L), methionine (M), and asparagine (N) were underrepresented in most positions. The enrichment statistical results showed that the sequence “N-X-T,” “N-X-S,” and “N-X-C” occurred at the highest frequency (Figure 6; Supplementary Table 5).

### 3.7. Functional enrichment of differentially expressed proteins in the protoscolex and adult worms of *Echinococcus granulosus*

**Figure 7:**
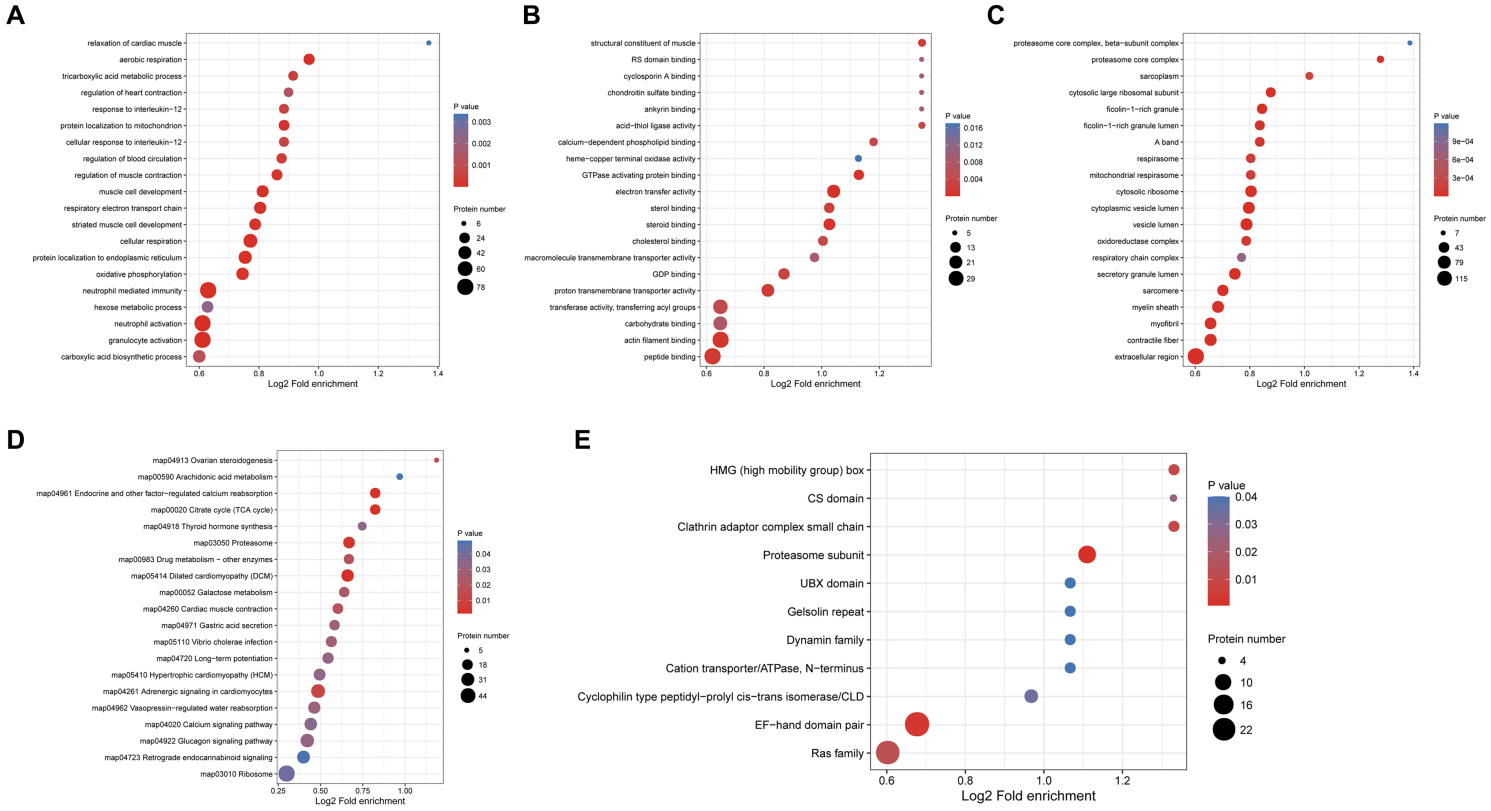
Functional enrichment of differentially expressed proteins in the protoscolex and adult worms of *E. granulosus*. **(A)** GO enrichment analysis of differentially expressed proteins for the biological process category. **(B)** GO enrichment analysis of differentially expressed proteins for the molecular function category. **(C)** GO enrichment analysis of differentially expressed proteins for the cellular component category. **(D)** KEGG enrichment analysis of differentially expressed proteins. **(E)** The protein domain enrichment analysis of differentially expressed proteins.

GO enrichment analysis of differentially expressed proteins in the protoscolex and adult worms showed that for the biological process category, 78, 78, 77, 50, and 43 differentially expressed proteins were enriched in neutrophil-mediated immunity, granulocyte activation, neutrophil activation, cellular respiration and protein localization to the endoplasmic reticulum (Figure 7A). In the molecular function category, 36, 35, 21, 20, and 16 differentially expressed proteins were enriched for peptide binding, actin filament binding, electron transfer activity, proton transmembrane transporter activity, and steroid binding, respectively (Figure 7B). In the cellular component category, 149, 63, 62, 61, and 60 differentially expressed proteins were enriched in the extracellular region, contractile fiber, vesicle lumen, cytoplasmic vesicle lumen, and myelin sheath, respectively (Figure 7C).

KEGG pathway enrichment analysis revealed that the differentially expressed proteins were significantly enriched in dilated cardiomyopathy (DCM; 27 proteins), proteasome (23 proteins), cardiac muscle contraction (18 proteins), endocrine and other factor-regulated calcium reabsorption (17 proteins), and citrate cycle (TCA cycle; 17 proteins; Figure 7D). Additionally, protein domain enrichment analysis showed that the differentially expressed proteins were significantly enriched in the EF-hand domain pair (26 protein domains), Ras family (24 protein domains), proteasome subunit (12 protein domains), and cyclophilin-type peptidyl-prolyl cis-trans isomerase/CLD (seven protein domains).

### 3.8. Functional enrichment of differentially expressed phosphorylated proteins in the protoscolex and adult worms of *Echinococcus granulosus*

**Figure 8:**
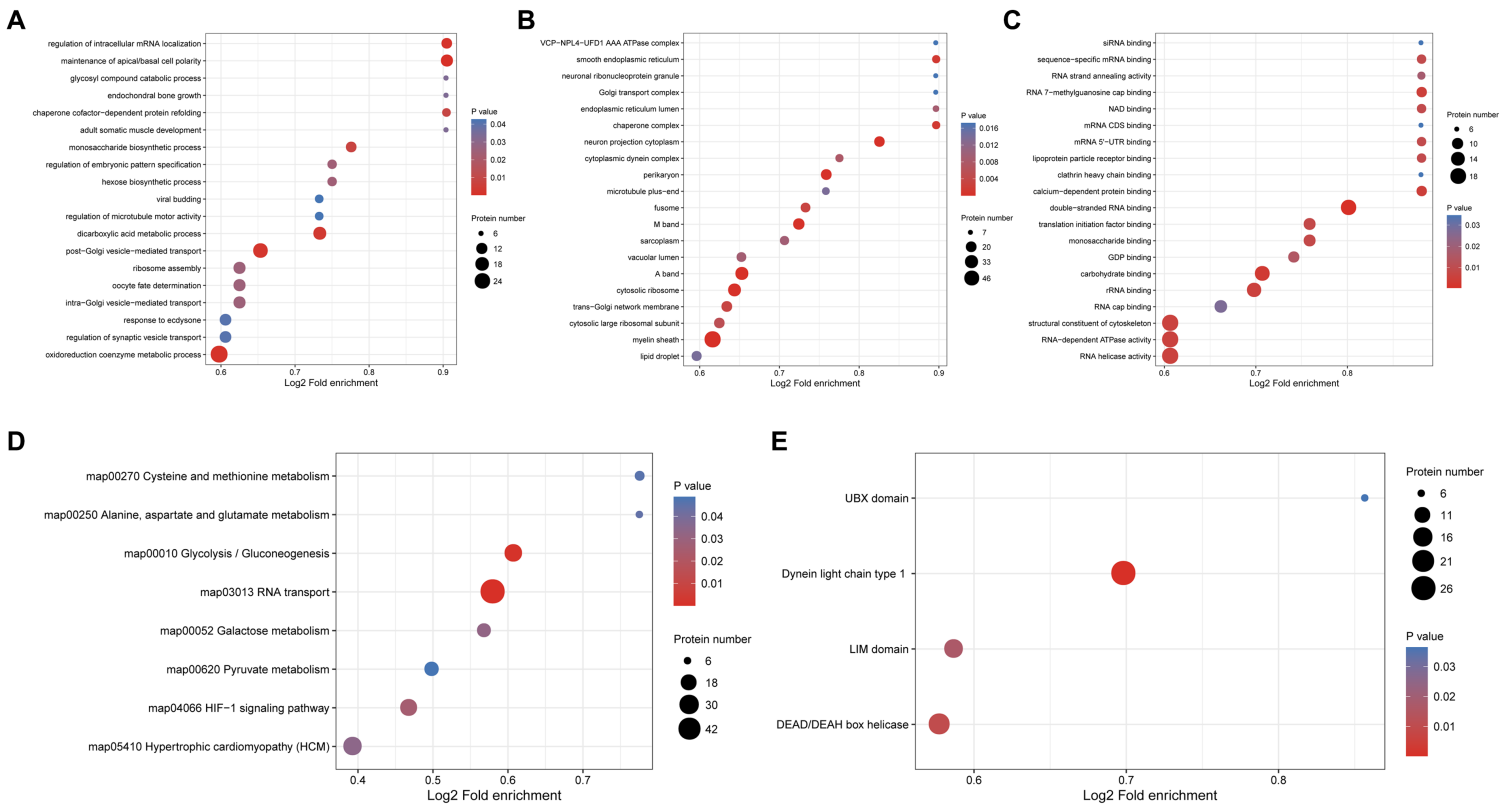
Functional enrichment of differentially expressed phosphorylated proteins in the protoscolex and adult worms of *E. granulosus*. **(A)** GO enrichment analysis of differentially expressed phosphorylated proteins for the biological process category. **(B)** GO enrichment analysis of differentially expressed phosphorylated proteins for the cellular component category. **(C)** GO enrichment analysis of differentially expressed phosphorylated proteins for the molecular function category. **(D)** KEGG enrichment analysis of differentially expressed phosphorylated proteins. **(E)** The protein domain enrichment analysis of differentially expressed phosphorylated proteins.

To explore the potential function of phosphorylated proteins in the protoscolex and adult worms of *E. granulosus,* GO analysis was performed for 1987 differentially expressed phosphorylated proteins. For the biological process category, 29, 21, 16, 14, and 14 phosphorylated proteins were enriched in oxidoreduction coenzyme metabolic process, post-Golgi vesicle-mediated transport, dicarboxylic acid metabolic process, maintenance of apical/basal cell polarity, and intra-Golgi vesicle-mediated transport, respectively (Figure 8A). In the cellular component category, 56, 32, 31, 23, and 20 proteins were mainly enriched in the myelin sheath, A band, cytosolic ribosome, M band, and perikaryon, respectively (Figure 8B). In the molecular function category, 19, 19, 19, 17, and 16 proteins were enriched in RNA helicase activity, RNA-dependent ATPase activity, structural constituents of the cytoskeleton, double-stranded RNA binding, and carbohydrate-binding, respectively (Figure 8C). KEGG analysis demonstrated that the differentially expressed phosphorylated proteins were mainly enriched in glycolysis/gluconeogenesis (23 proteins); cysteine and methionine metabolism (seven proteins); and alanine, aspartate, and glutamate metabolism (six proteins; Figure 8D). Furthermore, protein domain enrichment analysis indicated that the differentially expressed phosphorylated proteins were significantly enriched in the dynein light chain type 1 (26 protein domains), DEAD/DEAH box helicase (19 protein domains), and UBX domain (six protein domains; Figure 8E).

### 3.9. Functional enrichment of differentially N-glycosylated proteins in the protoscolex and adult worms of *Echinococcus granulosus*

**Figure 9:**
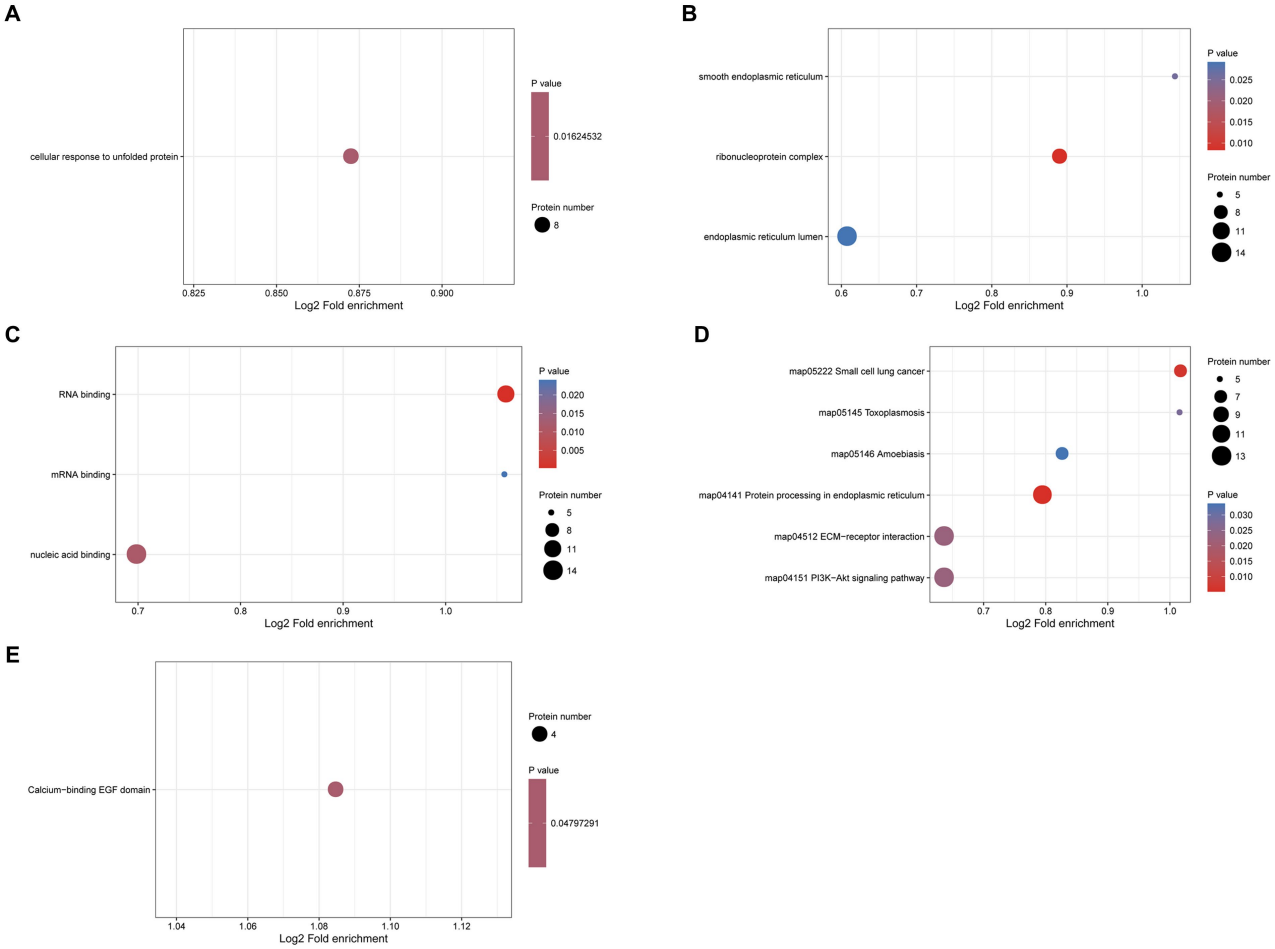
Functional enrichment of differentially expressed N-glycosylated proteins in the protoscolex and adult worms of *E. granulosus*. **(A)** GO enrichment analysis of differentially expressed N-glycosylated proteins for the biological process category. **(B)** GO enrichment analysis of differentially expressed N-glycosylated proteins for the cellular component category. **(C)** GO enrichment analysis of differentially expressed N-glycosylated proteins for the molecular function category. **(D)** KEGG enrichment analysis of differentially expressed N-glycosylated proteins. **(E)** The protein domain enrichment analysis of differentially expressed N-glycosylated proteins.

To explore the potential function of N-glycosylated proteins in the protoscolex and adult worms of *E. granulosus,* GO analysis was performed on 190 differentially expressed N-glycosylated proteins. In the biological process category, eight N-glycosylated proteins were enriched in the cellular response to unfolded proteins (Figure 9A). In the cellular component category, 14, nine, and five N-glycosylated proteins were enriched in the endoplasmic reticulum lumen, ribonucleoprotein complex, and smooth endoplasmic reticulum, respectively (Figure 9B). In the molecular function category, 14, 11, and five N-glycosylated proteins were enriched for nucleic acid, RNA, and mRNA binding, respectively (Figure 9C). KEGG pathway analysis showed that proteins were enriched in the PI3K-Akt signaling pathway, ECM-receptor interaction, protein processing in the endoplasmic reticulum, amoebiasis, and small-cell lung cancer (Figure 9D). Furthermore, protein domain enrichment analysis indicated that differentially expressed N-glycosylated proteins were significantly enriched in the calcium-binding EGF domain (Figure 9E).

### 3.10. Protein–protein interaction networks of the phosphorylated proteins and N-glycosylated proteins

The PPI network analysis results showed that for the phosphorylated proteins, a total of 516 nodes were identified in the PPI networks, 236 phosphorylated proteins were upregulated, and 280 phosphorylated proteins were downregulated in adult worms compared to the protoscolex (Supplementary Table 6). The term “hub” protein refers to a protein interacting highly with other proteins. For the PPI networks of phosphorylated proteins, the identified significant hub proteins included cell division cycle 5-related proteins, cyclin-G-associated kinase, elongation factor 1-gamma, DNA-directed RNA polymerase I and III subunits RPAC1 and Intersectin-1. For the N-glycosylated proteins, 55 nodes were identified in the PPI network. Nineteen N-glycosylated proteins were upregulated, and 36 N-glycosylated proteins were downregulated in adult worms compared to those in the protoscolex (Supplementary Table 7). Integrin beta, calnexin, endoplasmin, and basement membrane-specific heparan sulfate proteoglycan core proteins were the most significant hub proteins.

## 4. Discussion

This study systematically analyzed the proteome, phosphoproteome, and N-glycoproteome of PSCs and adult worms using a proteomic strategy. The results indicated that 693 proteins were upregulated and 756 were downregulated in adult worms compared to the PSCs. The previous research reported that 391 differentially expressed proteins were identified in adult vs. PSC, including 127 upregulated proteins and 264 downregulated proteins (22). Our study supplemented the data of differentially expressed proteins of PSCs and adult worms, and provided a new perspective for revealing the growth and development mechanism of *E. granulosus.* Among these differentially expressed proteins, a set of proteins were associated with the tricarboxylic acid cycle (TCA), such as malate, succinate, oxoglutarate, pyruvate, and lactate dehydrogenases. Some studies have reported that malate and lactate dehydrogenases may be important therapeutic targets for invasive parasites because they play critical roles in the progression and development of parasitic diseases (23, 24). These findings indicate that the TCA enzyme is widely expressed in the PSCs and adults, suggesting that carbohydrate metabolism might be the primary energy source during *E. granulosus* development, and that the key TCA enzymes might be important potential candidate targets for vaccines and novel drug research against CE. GO and KEGG pathway analyses showed that many differently expressed proteins were potentially responsible for male and female gamete generation, spermatogenesis and oocyte axis specification. To the best of our knowledge, *E. granulosus* has a well-developed reproductive system and undergoes sexual and asexual reproduction. The male and female reproductive organs germinate, mature, and degenerate in different proglottids (immature, mature, and gravid) (25). Some proteins related to fertilization were upregulated in adult worms, such as dihydrolipoyl dehydrogenase and ropporin-1-like, which are responsible for sperm capacitation, and CCT-beta and calnexin, which are associated with sperm-egg recognition (26, 27). Furthermore, a set of proteins related to male and female gamete generation was upregulated in the PSC of *E. granulosus*, such as LIM and SH3 domain proteins, which are associated with male gamete generation (28), and some important vaccine candidate antigens, such as major egg antigen, antigen EG13, FABP2, 14–3-3, and tetraspanin. Fatty acid-binding proteins (FABPs) are small intracellular proteins with a molecular mass of approximately 15 kDa that reversibly bind to fatty acids and other hydrophobic ligands (29). Because cestodes cannot synthesize fatty acids (30), FABPs, including other lipid-binding proteins that can facilitate lipid uptake from the host, have been proposed to be indispensable for these organisms. These findings suggest that FABPs could be used to develop antiparasitic vaccines and drugs (31). The 14–3-3 family of proteins is found in numerous eukaryotes, including parasites, and plays important roles in metabolic regulation, cell proliferation, and protein transport (32). The 14–3-3 protein is distributed in the extracellular vesicles and germinal layer and plays a vital role in the growth of *E. multilocularis*, indicating that it may regulate the invasive process of *E. multilocularis* (33). Therefore, the 14–3-3 protein is a potential candidate target for vaccine development.

Phosphoproteome analysis revealed that 2032 sites and 770 proteins were upregulated, and 2,993 sites and 1,217 proteins were downregulated in adult worms of *E. granulosus.* GO analysis results revealed that microtubule plus-end, microtubule binding, exocytosis, regulation of protein polymerization, adult somatic muscle development and regulation of cytokine production GO terms were enriched in more upregulated phosphoproteins in adult worms of *E. granulosus* compared to the PSC. Neuron projection cytoplasm, actin filament binding, dicarboxylic acid metabolic process, and chaperone cofactor-dependent protein refolding GO terms were enriched in downregulated phosphoproteins. KEGG analysis revealed that the differentially expressed proteins were mainly enriched in glycolysis/gluconeogenesis and amino acid metabolism, including alanine, aspartate, glutamate, cysteine, and methionine metabolism. Moreover, glycolysis/gluconeogenesis, phagosomes, RNA transport, SNARE interactions in vesicular transport, proteasome, pentose phosphate pathway, and phototransduction signal pathways were enriched in the upregulated phosphoproteins, while hypertrophic cardiomyopathy was downregulated. These findings indicate a difference in transcriptional and developmental regulation between adult worms and PSCs. Previous genome studies revealed that *E. granulosus* has complete pathways for glycolysis/gluconeogenesis (19). Phosphorylation occurs widely in key enzymes involved in glycolysis/gluconeogenesis, including fructose-bisphosphate aldolase, pyruvate dehydrogenase E1, phosphotransferase, triosephosphate isomerase, 2-phospho-D-glycerate hydrolase, glucose-6-phosphatase 3, phosphoglycerate kinase, phosphoenolpyruvate carboxykinase, pyruvate kinase, glyceraldehyde-3-phosphate dehydrogenase, L-lactate dehydrogenase, phosphoglucomutase, and dihydrolipoyl dehydrogenase. These findings suggested that phosphorylation is essential in glycolysis and gluconeogenesis in *E. granulosus*. Genome research on *E. granulosus* also showed that this cestode is capable of *de novo* synthesis of alanine, aspartic acid, and glutamic acid but lacks the capability for *de novo* synthesis of pyrimidines, purines, and most amino acids (19). Compared to *S. mansoni* (34), the capacity to synthesize amino acids is further reduced, with serine and proline biosynthesis enzymes absent in some tapeworms (35). The present study indicated that phosphorylation events occur in some enzymes associated with alanine, aspartate, glutamate, cysteine, and methionine metabolism, including glutamate synthase, glutamate dehydrogenase, aspartate aminotransferase, succinate-semialdehyde dehydrogenase, adenylosuccinate synthetase, S-adenosylmethionine synthase, and L-threonine 3-dehydrogenase, suggesting that phosphorylation is important for amino acid metabolism in *E. granulosus.*

In the N-glycoproteome analysis, we found that 90 sites and 64 proteins were upregulated, and 126 sites and 64 proteins were downregulated in the adult worms of *E. granulosus*. GO analysis revealed that membrane-enclosed lumen, RNA binding, and nucleic acid transport GO terms were enriched in the upregulated N-glycoproteins in adult worms of *E. granulosus* compared to those in the PSC. However, the GO terms lumenal side of the endoplasmic reticulum membrane, amide binding, peptide metabolic process, and epidermal cell differentiation were enriched in the downregulated N-glycoproteins. KEGG analysis revealed that the significantly differentially expressed N-glycoproteins were enriched in protein processing in the endoplasmic reticulum, small cell lung cancer, the PI3K-Akt signaling pathway, and the ECM-receptor interaction signal pathway. Among these significantly differentially expressed N-glycoproteins, calreticulin was involved in many GO terms. Calreticulin is a highly conserved Ca^2+^−binding protein found in all cells except erythrocytes in higher organisms; it mediates the phagocytosis of apoptotic cells, regulates autoimmune responses, inhibits angiogenesis and tumor growth, and participates in ER calcium storage, signal transduction, regulation of gene expression, cell adhesion, and other biological functions (36). Several protozoa and multicellular parasites have been found to express calreticulin on the body surface and even secrete calreticulin *in vitro*, which plays an important role in parasite–host interactions; in particular, it is closely related to parasite infection and host immune response (37). Considering the important role of calreticulin in the interaction between parasites and their hosts, we speculate that it may be a potential vaccine target against *E. granulosus*. Serine protease inhibitors are potent inhibitors of chymotrypsin and neutrophils, which bind calcium during local inflammation and reduce neutrophil infiltration. Furthermore, it may be involved in host immune evasion by inhibiting neutrophils and cathepsin (38). Serine protease inhibitors from *E. granulosus* inhibit the production of inflammatory cytokines and macrophage proliferation of macrophages (39). Other studies have shown that serine protease inhibitors of *Fasciola hepatica* can protect the parasite from the hydrolysis of harmful proteins (such as chymotrypsin) produced by the host during invasion (40). Another important and significantly differentially expressed N-glycoprotein family was the tetraspanins, which were verified as expansion families in platyhelminths and are likely components of the host-pathogen interface (41). Some studies have reported that tetraspanins, which are released by helminths within hosts, are part of extracellular vesicles (42). Other studies have found that tetraspanins can bind to the Fc domain of host antibodies (43) or stimulate the host to produce specific antibodies and reduce the parasite burden (44–47).

In summary, this study employed phosphorylation and N-glycosylation-specific enrichment techniques, coupled with advanced mass spectrometry and computational data analyses, to establish the initial global phosphoproteome and N-glycoproteome of both the PSCs and adult worms of *E. granulosus*. The findings of this research offer significant insights into the expression patterns of *E. granulosus* and present a novel approach to understanding the involvement of protein phosphorylation and N-glycosylation in the developmental processes of *E. granulosus*.

## Data availability statement

The datasets presented in this study can be found in online repositories. The names of the repository/repositories and accession number(s) can be found below: PXD043166 (ProteomeXchange).

## Author contributions

ZW: Funding acquisition, Methodology, Project administration, Writing – original draft. JM: Resources, Writing – original draft. YZ: Resources, Methodology, Writing – review & editing. YS: Formal Analysis, Writing – original draft. XB: Supervision, Writing – review & editing. XJ: Formal Analysis, Writing – review & editing.

## Footnotes

^1^http://www.ebi.ac.uk/interpro/
